# Antimicrobial Treatment of Polymeric Medical Devices by Silver Nanomaterials and Related Technology

**DOI:** 10.3390/ijms18020419

**Published:** 2017-02-15

**Authors:** Markéta Polívková, Tomáš Hubáček, Marek Staszek, Václav Švorčík, Jakub Siegel

**Affiliations:** 1 Department of Solid State Engineering, University of Chemistry and Technology Prague, Prague 166 28, Czech Republic; polivkoa@vscht.cz (M.P.); marek.staszek@vscht.cz (M.S.); vaclav.svorcik@vscht.cz (V.Š.); 2 Institute of Hydrobiology, Biology Centre of the AS CR, Ceske Budejovice 370 05, Czech Republic; hubacektom@gmail.com

**Keywords:** antimicrobials, medical devices, nanostructures, polymers, modification, biocompatibility

## Abstract

Antimicrobial biocompatible polymers form a group of highly desirable materials in medicinal technology that exhibit interesting thermal and mechanical properties, and high chemical resistance. There are numerous types of polymers with antimicrobial activity or antimicrobial properties conferred through their proper modification. In this review, we focus on the second type of polymers, especially those whose antimicrobial activity is conferred by nanotechnology. Nanotechnology processing is a developing area that exploits the antibacterial effects of broad-scale compounds, both organic and inorganic, to form value-added medical devices. This work gives an overview of nanostructured antimicrobial agents, especially silver ones, used together with biocompatible polymers as effective antimicrobial composites in healthcare. The bactericidal properties of non-conventional antimicrobial agents are compared with those of conventional ones and the advantages and disadvantages are discussed.

## 1. Introduction

In recent decades, biocompatible polymers have become wide-spread materials in medicinal technology. However, it was found that there is a risk associated with their application; their long-term use often leads to bacterial colonisation, biofilm formation and development of hospital-acquired infections, so-called nosocomial infections [1]. It is difficult to treat those infections, and when they fully develop they are accompanied by considerably unpleasant symptoms, such as fever followed by purulent drainage and overwhelming sepsis, and might lead to death [2]. The most widely types of nosocomial infections are: catheter-related bloodstream infection, ventilator-associated pneumonia, surgical site infection and catheter-associated urinary tract infection, which are often caused by Staphylococci, Pseudomonads and *Escherichia coli* [3,4]. 

In an attempt to reduce the frequency of such infections and substantially decrease morbidity and mortality, postoperative antibiotic therapy was the first-choice method to fight against these infections. Nevertheless, problems associated with their long-term use begun to appear. The major problem is antimicrobial resistance, especially the acquired type. Of greatest concern are methicillin- and vancomycin-resistant *Staphylococcus aureus*, vancomycin-resistant Enterococcus, and extended-spectrum β-lactamase-producing Gram-negative bacilli. Due to these disadvantages of conventional antibiotics, new methods of prevention are being researched [5,6].

One effective way to prevent nosocomial infections is by using antimicrobial-biocompatible polymers instead of common ones. This is a gentle, safe and non-conventional method to prevent biofilm formation. Two types of antimicrobial polymers might be used in this case: (i) polymers that exhibit antimicrobial activity by themselves; or (ii) ones whose antimicrobial properties are conferred through their proper modification [7]. The first group represents polymers and co-polymers, i.e., cationic macromolecules (with positively charged active groups, including biguanide, quaternary ammonium, pyridinium or phosphonium salts, and also cyclic *N*-halamine), polyionenes (with positively charged nitrogen atoms), which could be natural or synthetic, and also polymers or oligomers that can mimic the biological activity of the antimicrobial natural host-defense peptides (synthetic poly(phenylene ethynylenes), polynorbornenes, and polymethacrylates) [8,9,10]. The second group are antimicrobial-treated polymers with added organic and/or inorganic antimicrobial agents. These antimicrobial agents might be (i) incorporated throughout the volume of polymeric matrix; or (ii) form coatings on the polymeric surface. Their presence causes the reduction of initial microbial adhesion to surfaces and inactivation of microorganisms already adherent to a surface [11]. Nanotechnology is a promising tool for the preparation of antimicrobial-treated polymers. Nanostructured noble metals with considerable antimicrobial activity, most commonly silver in its various forms (nanoparticles, nanolayers, nanowires, etc.), might be used effectively [12,13,14,15]. 

Generally, the exploitation of nanotechnology in the development of antimicrobial polymeric materials is experiencing increasing interest; for this reason, we focus on this group of materials in the following sections. Possible types of highly effective nanostructures and techniques for their preparation are described, and the advantages and disadvantages in comparison with conventional antibiotics are investigated.

## 2. Polymeric Medical Devices Related to Nosocomial Infections

**Figure 1:**
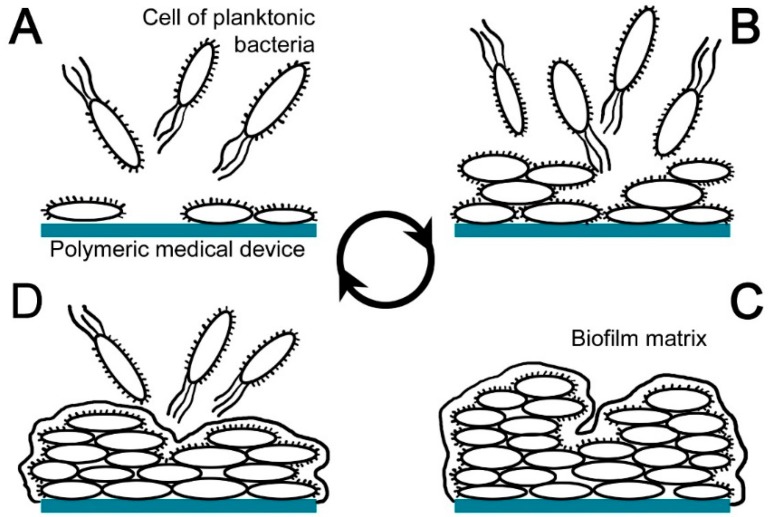
Phases of biofilm formation: (**A**) attachment; (**B**) expansion; (**C**) maturation; (**D**) dispersion.

Nowadays, a broad spectrum of nosocomial pathogens is known and their main sources are the skin of the patients and/or clinicians during surgical interventions. The transmission of pathogens via the hands of healthcare workers is often through incision channels to device surfaces, followed by bacterial colonization and biofilm formation (Figure 1). Biofilm is a community of microorganisms attached to a surface and surrounded by polysaccharides secreted by biofilm cells. A polysaccharide capsule is formed around biofilm cells and together with proteins, nucleic acids and lipids produces a coating layer around the clusters of cells, a so-called matrix. Biofilm provides mechanical stability, mediates its adhesion to the surface and forms a polymeric spatial network, which attaches the individual planktonic bacterial cells to the surface. Biofilm thus behaves like a single organism. The mechanism of its formation is not fully understood yet; however, it contains four individual phases: (i) attachment—at first initial, and then irreversible, using specialized tail-like structures; (ii) expansion—bacterial growth and division, formation of dense mat of many layers, using specific signals to communicate with each other; (iii) maturation—a quorum (minimum number of members), bacterial secretion of sugar substances acting as a glue, formation of mushroom-shaped structures using specialized tail-like structures; and (iv) dispersion—part of biofilm release free floating bacteria for further colonization.

Biofilm formation may lead to haematogenous spread, and the development of both local and systemic infections. The type of nosocomial infection is then determined by the type and location of medical device. Examples of medical devices associated with the occurrence of nosocomial infections are shown in Table 1 [16,17,18].

Once the biofilm is formed on the surfaces of polymeric medical devices, one can observe the biodeterioration of these materials related to their structure and function after a certain period of time. Polymeric materials are damaged by biofilms in various ways, such as by:

- coating of the material surface, masking of its surface properties and contaminating surrounding media (body fluids);
- leaching out of additives and monomers originating from polymer by microbial degradation;
- enzymatic and/or radical attack of polymer and additives, which causes embrittlement of the material and the loss of its mechanical stability;
- accumulation of water and penetration of microbial filaments into the polymer, which results in increased conductivity of material and formation of swelling;
- excretion of lipophilic microbial pigments, which leads to a colour change in the polymer.

Direct and indirect damage of the polymeric material of medical device caused by these five mechanisms is irreversible and often causes the failure of treatment [19,20].

The most common types of nosocomial infections caused by Staphylococci, i.e., *Staphylococcus epidermidis* are urinary tract infections, pneumonia, septicaemia and surgical wound infections [18,21]. Enterococci, such as multiresistant *Enterococcus faecium*, have already been isolated from cultures of blood, urine, or surgical wound specimens from eight additional patients [22]. Enterobacteriaceae, e.g., *Enterobacter sakazakii*, may cause serious diseases like meningitis, bacteremia and colonization of neonates [23]. Immunocompromised patients, for example patients with acute lymphocytic leukaemia, are at particularly high risk of the development of candidemia; colonization of stool by *Candida species* [24]. Generally, all types of nosocomial infections are difficult to treat. One of the most important problems is microbial resistance to conventional antibiotics [18,19,20,21,22,23,24].

## 3. Methods of Antimicrobial Treatment of Polymeric Medical Devices

Adhesion of microorganisms on implanted medical devices or damaged tissue causes a formation of a thin layer, a so-called biofilm, and leads to persistent infections. The resistance of bacteria to conventional antibiotics during its formation significantly contributes to the chronicity of nosocomial infections. The mechanisms of resistance to conventional antibiotics might be divided into two groups: (i) primary—natural, relates to bacterial species that are outside the range of action of antibiotics, do not contain a structure on which antimicrobial agents affects; and (ii) secondary—acquired, the result of evolution of bacterial genome and selection pressure of surroundings. This is caused by the mutation of a gene in a bacterial chromosome or the acquisition of a gene from another bacterium (resistant to the antimicrobial agent).

**Figure 2:**
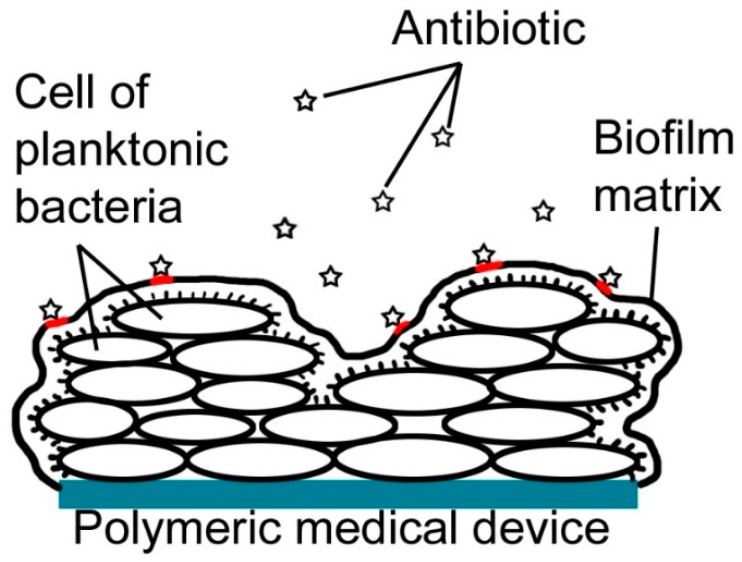
Bacterial resistance of biofilm. Red underlines refer to the area of antibiotic/biofilm matrix contact. Biofilm matrix prevents antibiotics to affects directly the cells of planktonic bacteria.

In biofilms, bacterial resistance develops on a multicellular level (Figure 2). The matrix, containing polysaccharide capsule, proteins, nucleic acids and lipids, secretes a glue-like substance that protects bacteria against the outside environment and shields them against the action of antibiotics. Since the biofilm formation is associated with the chronic nature of the infections, resistance to conventional antibiotic therapy represents the major problem with this method of treatment [25,26,27]. Various methods of antimicrobial treatment of medical devices have already been used to fight nosocomial infections and subsequent bacterial resistance (Table 2). As time went on, more and more elaborated antimicrobial coatings were investigated.

**Figure 3:**
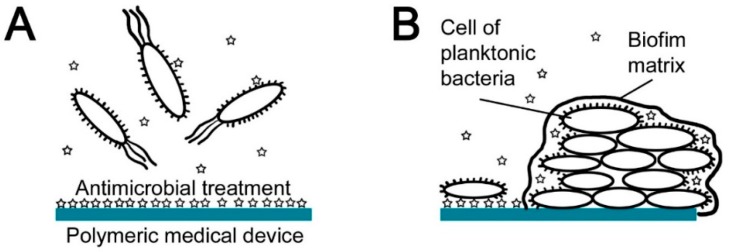
Effects of antimicrobial treatment of polymeric medical devices: (**A**) prevention of bacterial adhesion; (**B**) inhibition of bacterial colonization (preclusion of biofilm formation).

Antimicrobial treatment of polymeric medical devices can be accomplished by various means, which are naturally accompanied by some advantages and disadvantages. Conventional antibiotics can, like other treatment methods, be used effectively as a two-phase-acting treatment (Figure 3). 

### 3.1. Conventional Antibiotics and Antiseptics

Instead of common washing of medical devices by antibiotics, conventional antibiotics were bonded by chemical bond with a material of medical device or polymeric matrix as a reservoir of antibiotics to attain the sustained release of antimicrobial agent and prolonged effect. In the text bellow, one can see some examples of the use of conventional antibiotics in their various forms. 

Chemaly et al. [28] examined long-term silicone catheters coated with minocycline and rifampin as a prophylactic strategy of intravascular catheter-related infections and compared them to non-coated ones. They found that this antimicrobial coating was effective against methicillin-resistant *Staphylococci* and Gram-negative bacilli. Jose et al. [29] added covalently bonded vancomycin to titanium beads to create a self-protecting implant with increased biocompatibility due to the presence of titanium. Because the ionization of silver metal is proportional to the surface area of the particle exposed, silver sulfadiazine ionic bonded to medical devices could be an effective way to prevent biofilm formation. Fox et al. [30] found that sulfadiazine did not act as an antibacterial agent in low concentrations, but exhibited specific synergism in combination with subinhibitory levels of silver sulfadiazine. The efficacy of silver sulfadiazine was caused by its slow and steady reactions with serum and other sodium chloride-containing body fluids, which permits the slow and sustained delivery of silver ions into the wound surroundings. Elsner et al. [31] investigated the antibacterial efficacy of novel dressings based on a polyglyconate mesh coated with a porous poly-(dl-lactic-*co*-glycolic acid) matrix loaded with the antibiotic drugs ceftazidime and gentamicin. The dressings resulted in 99.99% inhibition effect of *Pseudomonas aeruginosa* and *Staphylococcus albus*. Persistent bacterial inhibition zones around the dressing material indicated a long-lasting antimicrobial effect.

Improved techniques for the antimicrobial treatment of medical devices by conventional antibiotics were successfully discovered; however, these approaches represent only one possible way to fight nosocomial infection. Another way to reduce the disadvantages of conventional antibiotics is the use of antiseptics.

To overcome the problem of bacterial resistance to antibiotics, the impregnation of medical devices to prevent biofilm formation by antiseptics might be used. However, due to the low efficacy of antiseptics and low patient tolerance, only short-term usage is possible [41]. For this reason, Sheng et al. [32] studied patient tolerance and the antimicrobial efficacy of a novel antiseptic central venous catheter, made of polyurethane and impregnated with chlorhexidine. They found that chlorhexidine impregnation reduced the risk of catheter-related infections caused by Gram-positive cocci and fungi and no adverse reactions such as hypersensitivity or leukopenia were found. This antiseptic coating was safe and had less risk of colonization of bacteria and fungi than standard catheters; however, more efficient and safer ways of antimicrobial treatment have since been found.

### 3.2. Non-Antibiotics 

Due to the disadvantages of the abovementioned antimicrobial treatments of medical devices, the current trend is for the development of non-conventional antimicrobial agents, such as non-antibiotic and/or noble-metal-inspired techniques, instead of employing conventional antibiotics and antiseptics.

**Figure 4:**
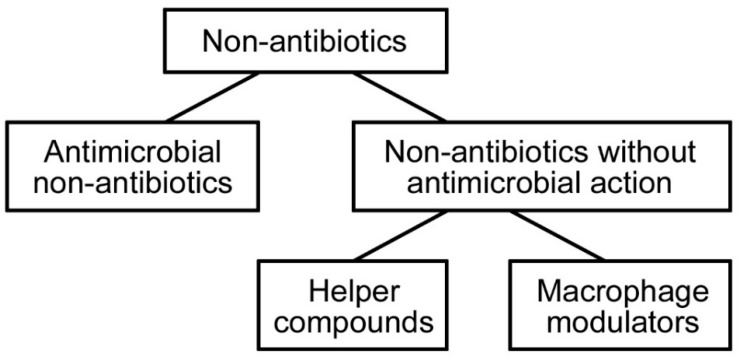
Scheme of the division of non-antibiotics.

Non-antibiotics are synthetic, non-chemotherapeutic compounds that are used in the management of pathological conditions of a non-infectious aetiology. They are divided into two groups with distinctly different effects (see Figure 4). The first group, antimicrobial non-antibiotics, are those that exhibit direct antimicrobial activity. The second group consists of two subgroups, one of which, helper compounds, alters the permeability of the microorganism to a given antibiotic; the second, macrophage modulators, enhances the killing activity of macrophages that have phagocytosed the microorganism. Due to the phenomenon of multidrug resistance, especially of Gram-negative bacteria, the use of non-antibiotics may effectively fight against nosocomial infections. Non-antibiotics eliminate natural resistance to specific antibiotics (reversal of resistance) and exhibit strong activity against multidrug-resistant species [26,42]. Wainwright et al. [33] dealt with this subject matter. They examined the bactericidal activity of methylene blue and its derivatives against vancomycin-resistant *Enterococcus faecalis* and *Enterococcus faecium*. They found that they are suitable for the disinfection of sites of drug-resistant infection, such as burn wounds, colonized breathing tubes, drains and catheters, and that the use of methylene blue derivatives may overcome the problem of drug-resistant microbes.

The next stage of the development of advanced antimicrobials in this category represents a combination of non-antibiotics with conventional antibiotics, which gives rise to a synergic antimicrobial effect. The advantage of this synergic combination is the use of a drug that has no useful antimicrobial activity at the concentration used, but its presence increases the activity of the second antibiotic to which the organism was previously resistant. Another advantage is that whenever non-antibiotic components exhibit potential toxicity alone, combining them with conventional antibiotics may lead to their safe use due to the significantly reduced concentration needed to produce the desired antimicrobial effect [26,42]. These advantages of the combination of non-antibiotics with conventional antibiotics confirm the study of Rahbar et al. [34]. They evaluated the antimicrobial and resistance-reversal activity of phenothiazine derivatives in combination with vancomycin against vancomycin-sensitive *Enterococcus faecalis*, vancomycin resistant *E. faecalis* and vancomycin-resistant *E. faecium*, originating from human infections. They found that phenothiazines, especially chlorpromazine, thiethylperazine and promethazine, could considerably reduce the resistance of the abovementioned strains to vancomycin in vitro.

### 3.3. Noble Metals

Another important and effective antimicrobial treatment of polymeric medical devices is the use of noble metals, especially silver. Silver has a long history as an antimicrobial agent in medicine. It has been used in various healthcare areas, such as wound care, surgical instruments, reconstructive orthopaedic surgery, bone prostheses, cardiac devices and catheters. The use of silver to reduce the risk of nosocomial infection has gained importance in recent days [43,44].

The antimicrobial effect of silver and silver compounds is proportional to the quantity of released silver ions and their availability to interact with bacterial and fungal cell membranes. Each silver source, from inorganic silver compounds (e.g., AgNO_3_ [45]), via silver complexes (*N*-heterocyclic carbene–silver complexes [46]), to nanostructured silver (e.g., nanoparticles [47], nanolayers [48] and nanowires [49]), should release silver ions and therefore might be effective in fighting pathogenic microorganisms. Due to its unique advantages, such as low toxicity in the human body and minimal risk in clinical exposure by inhalation, ingestion, dermal application or through the urological or haematogenous route, silver in its various forms has considerable potential in the healthcare industry [43,44]. 

Incorporation of silver, in the form of salts, ions and complexes, is the gold standard in the antimicrobial treatment of polymeric medical devices. Balazs et al. [35] incorporated silver ions using silver salt (AgNO_3_) into an endotracheal tube, made from medical-grade polyvinylchloride (PVC), to protect it against *Pseudomonas aeruginosa* adhesion and colonization. They found that surface modification incorporating silver ions into PVC was effective in the reduction of bacterial colonization of medical devices. Becker et al. [36] used silver iontophoresis to electrically generate silver ions for the preparation of a silver–nylon dressing preventing orthopaedic infections. In this case, silver ions acted as a strong antibacterial agent by in vitro testing. Panzner et al. [37] investigated *N*-heterocyclic carbine–silver complexes as a potentially new effective antimicrobial agent against skin and soft tissue, respiratory, wound, blood and nosocomial infections. They were synthesized and tested against the group of biosafety level 3 bacteria *Burkholderia pseudomallei*, *Burkholderia mallei*, *Bacillus anthracis*, methicillin-resistant *Staphylococcus aureus* (MRSA) and *Yersinia pestis* to establish their antimicrobial efficacy against virulent and antibiotic-resistant bacteria associated with cystic fibrosis lung infections. They found that, in the case of biofilm organisms of *B. anthracis*, these complexes proved more effective than the currently used clinical antibiotics ciprofloxacin and doxycycline. In the case of mature cultures of planktonic *B. anthracis* and MRSA, they were more effective than the aminoglycoside antibiotic gentamicin. 

The proven antimicrobial activity and low cytotoxicity of silver led to the development of such materials by nanotechnology. In light of the last decade’s research, nanostructured silver is an excellent candidate for the antimicrobial treatment of biocompatible polymers in the fight against nosocomial infections [49,50]. As well as silver, other noble metals such as gold [38,47], copper [39] and palladium [40,51] exhibit strong antimicrobial effects. We can encounter manifold types of nanostructures in this case, e.g., nanolayers, nanoislands and nanowires; however, the type that is usually used is nanoparticles. 

In the text below, we provide some examples from the literature addressing the use of noble metal nanoparticles (Ag, Au, Cu and Pd) with a strong antibacterial response. It is apparent that all of those variously nanostructured metals are suitable candidates for antimicrobial coatings of polymeric medical devices. 

Roe et al. [12] functionalized a plastic catheter by silver nanoparticles (AgNPs) and tested its antimicrobial response. Model pathogens *Escherichia coli*, Enterococcus, *Staphylococcus aureus*, coagulase-negative staphylococci, *Pseudomonas aeruginosa* and *Candida albicans* were chosen as the most commonly involved microorganisms in catheter-related infections. Silver-coated catheters showed significant in vitro antimicrobial activity and prevention of biofilm formation. Li et al. [38] prepared functionalized gold nanoparticles (AuNPs) as an antimicrobial agent against multidrug-resistant pathogenic bacteria. They found a strong antimicrobial response and high biocompatibility of AuNPs, the usage of which slowed the development of bacterial resistance. Due to these advantages, gold nanoparticles can be used in long-term applications in healthcare. Ben-Sasson et al. [39] functionalized polyamide membrane by copper nanoparticles and examined it against *P. aeruginosa* and *E. coli*. They found that the antibacterial activity and relatively low cost of copper made it an attractive antimicrobial agent. Adams et al. [40] studied the inhibitory effects of palladium nanoparticles (PdNPs) compared with Pd^2+^ ions toward Gram-negative *E. coli* and Gram-positive *S. aureus* bacterial strains. They found that PdNPs have a strong potential for antimicrobial applications, especially against Gram-positive bacteria; PdNPs were more effective than Pd^2+^ ions against *S. aureus*, while *E. coli* required higher concentrations and longer exposure times before the inhibitory effect of PdNPs became evident. 

The unique potential of nanostructured silver to kill infectious microorganisms currently makes it one of the most powerful antimicrobial agents. Therefore, the rest of our review will be devoted to various types of silver nanostructures, the mechanism of their antimicrobial action, and techniques for their preparation.

## 4. Nanostructured Silver

In recent years, the term nanomaterial has penetrated into the human consciousness. One of the most common types of nanomaterials is nanostructured silver, which, due to its well-known antimicrobial activity, has found its major application in medicine. In the nanoscale, silver may occur in several forms, predominantly as nanoparticles, nanowires, and last but not least as nanolayers. The most often used silver nanomaterial is nanosilver in the form of silver nanoparticles, formed by clusters of silver atoms [52]. The most significant advantages of nanostructured silver for the use in medicine are its remarkable physical, chemical and biological properties, including unique antimicrobial activity [53]. Examples of medical applications of silver nanoparticles are summarized in Table 3.

### 4.1. Mechanism of Antimicrobial Action

**Figure 5:**
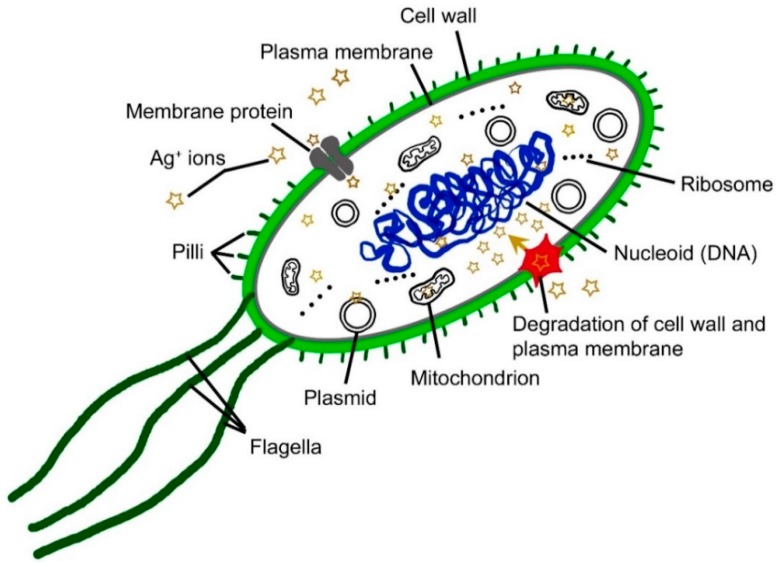
Multifaceted mechanisms of the antibacterial action of silver ions.

Even though the mechanism of antimicrobial action of nanostructured Ag has not been fully clarified, it is well known that silver nanoparticles of various sizes and shapes, after their partial oxidation, release Ag^+^ ions, which act as the major bactericidal agent. Silver ions interact with four main components in bacterial cells (Figure 5): the cell wall, plasma membrane, bacterial DNA and proteins (i.e., specific enzymes involved in vital cellular processes, such as the electron transport chain). Silver ions cause the degradation of the peptidoglycan cell wall and cell lysis, preventing bacterial propagation. These ions penetrate to the cell interior, where they bind to DNA bases. DNA condenses and loses its replication ability, thereby preventing bacterial reproduction via binary fission. They may also cause chromosomal aberrations. In the cell interior, silver ions may also cause mitochondrial dysfunction and may desaturate ribosomes, thereby inhibiting protein synthesis and causing the degradation of plasma membrane. This multifaceted mechanism of antibacterial action is the main factor that causes low bacterial resistance against silver [52,54].

Silver ions were also described as efficient antibacterial agents in the form of nanostructured silver/polymer composites, in which their long-term release is necessary to be successfully used as an effective antimicrobial coating of polymeric medical devices [55]. For this reason, the evaluation of the concentration of released Ag^+^ ions and their dependence on time are important parameters to observe. The concentration of released silver ions must then reach the value of minimum inhibitory concentration (MIC) to be effective in bacterial inhibition. The rate of release depends on the chemical form of silver (e.g., AgNPs), particle size, surface functionalization and particle crystallinity. Last but not least, the temperature and nature of the immersion medium, such as the presence of salts or biomolecules, are key factors [56].

The literature mentions minimum inhibitory concentrations of silver effective against single-cell organisms ranging from 0.1 to 20 mg·L^−1^. For example, MIC for Gram-negative bacteria *E. coli* typically ranges from 3.50 (Ag^+^) to 13.02 mg·L^−1^ (AgNPs), and for Gram-positive *S. epidermidis* it is ca. 6.25 mg·L^−1^ for AgNPs (depending on the size and shape of these structures); unfortunately, for Ag^+^ no reference value was reported [57]. On the other hand, Jung et al. [58] reported that silver ions showed strong bactericidal effects against *S. aureus* and *E. coli* already in the concentration of one-tenth of a ppm. The cells of *S. aureus* treated with tens of ppm silver ion solution for 2 h underwent lysis, which resulted in the release of their cellular contents into the surrounding environment. The *E. coli* cells treated with silver ions showed aberrant morphology: they were cracked and ruptured. It follows that not only the concertation of silver ions, but also other factors such as the contact of bacteria with metal surface, should be taken into account. The values of concentrations of silver ions released from treated polymeric medical devices also differ depending on their type; however, MICs remain unchanged in the order [59]. Jansen et al. [60] calculated the MIC of silver ions incorporated in a polyurethane catheter against *E. coli* and *S. epidermidis* as 10.62 mg·L^−1^ and 1.33 mg·L^−1^, respectively. Osińska-Jaroszuk et al. [61] studied MIC for silver ions released from an InterGard Silver^®^ (MAQUET Holding B.V. & Co. KG, Rastatt, Germany) vascular prosthesis made from polyester and collagen. They found that the value of MIC is 10 mg·L^−1^ for *E. coli* and 5 mg·L^−1^ for *S. aureus*.

For effective prevention of nosocomial infections, the lifetime of antimicrobial coatings, after which they lose antimicrobial activity (reach MIC), is an important parameter to study, and the value of MIC must be eluted constantly. For example, the average duration of placement of a central venous catheter is 10–14 days [62]. The problem occurs in the case of conventional antibiotic treatment. Trooskin et al. [63] give the lifetime of benzylpenicillin treatment of polytetrafluoroethylene catheter as 24 h against *S. aureus*. Jansen et al. [64] found the teicoplanin coating of a polyurethane catheter to be effective against *S. epidermidis* for 72 h. However, longer-term effects have already been observed: Raad et al. [65] studied rifampin and minocycline coatings of polytetrafluoroethylene catheter against various bacteria (25 days); and Hampl et al. [66] investigated silicone catheter coated by rifampin (60 days). Unlike conventional antibiotics, the duration of the antimicrobial effect of silver-impregnated catheters is reported to be approximately 48 days [67]. Bassetti et al. [68] compared the antimicrobial lifetime of Arrowgard Blue^®^ (Teleflex, Morrisville, NC, USA) central venous catheters impregnated by chlorhexidine (C) and silver sulfadiazine (S) with the same one with a three times larger amount of C and the same amount of S. The experiments were conducted against *S. aureus*. After 34 days, the second type of catheter still produced inhibition zones. In contrast, the antimicrobial activity of the first type of catheter was completely lost after 20 days. Samuel et al. [1] studied the duration of silver release from polyurethane and silicone catheters impregnated with silver nanoparticles. The duration of silver release was investigated with continuous rinsing of the material with physiologic saline. No concentration change of released silver was observed for 370 days.

In recent days, the problem with the genotoxicity of nanostructured silver following exposure to mammalian cells is coming into light. Braydich-Stolle et al. [69] investigated the ability of different types of silver nanoparticles to damage a mouse spermatogonial stem cell line in the male germline in vitro. They found that silver nanoparticles reduced mitochondrial function and increased reactive oxygen species (ROS) generation and membrane leakage of mammalian germline stem cells. It is well known that ROS cause DNA damage, such as a multitude of oxidized base lesions, abasic sites, and single- or double-strand breaks. They may also induce apoptosis (cell death). For these reasons, silver nanoparticles could be potentially cytotoxic and/or mutagenic [70]. The damage of DNA can be easily observed in the pathway of p53 protein responsible for DNA repair and apoptosis response to maintain genomic stability. Ahamed et al. [71] studied the DNA damage and apoptosis induced by silver nanoparticles through the p53 pathway in mammalian cells. Their results demonstrated p53 protein expression, DNA double strand breakage and apoptosis responses in both investigated mouse embryonic stem (mES) and mouse embryonic fibroblasts (MEF) mammalian cells. The molecular mechanisms of nanoparticle toxicity are still poorly understood and this problem should not be underestimated.

### 4.2. Types of Silver Nanostructures 

Nanostructures are defined as objects having at least one dimension between 1 and 100 nm. Depending on the number of directions in which they meets this condition, they can be divided into three groups: 0D—all three dimensions are on nanoscale (nanoparticles), 1D—two dimensions on nanoscale (nanowires), and 2D—one dimension on nanoscale (nanolayers).

Nowadays, there are various types of nanostructures available to prepare (nanoparticles, nanowires, nanolayers, nanofibres, nanorods, nanocubes, nanosheets, nanofoam, etc. [72,73]). Given the large number of possible structural forms, the first three were chosen for further description.

#### 4.2.1. Silver Nanoparticles

**Figure 6:**
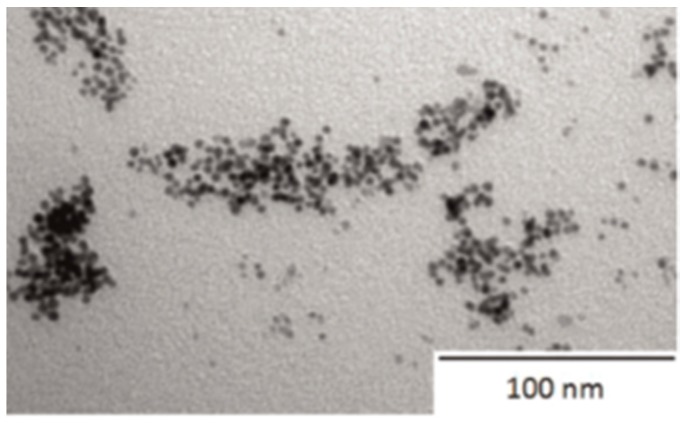
Transmission electron microscopy (TEM) image of AgNPs sputtered into glycerol [74].

A nanoparticle is a type of 0D nanostructure of defined size ranging from 1 to 100 nm, formed by clusters of silver atoms. In Figure 6 one can see spherical AgNPs (size of 2.5 ± 0.6 nm) prepared by the direct sputtering method into glycerol. Silver exhibits excellent antibacterial activity and low cytotoxic potential—a combination of the most suitable properties for medical applications as the antibacterial coatings of polymeric medical devices [47,74]. The desired properties of nanoparticles generally depend not only on their size, but also on the specific shape. If synthesized nanoparticles do not exhibit the expected properties in all, these problems can be easily overcome by the use of nanotechnology, which can nowadays effectively control those material parameters. Silver could be processed into monodisperse nanoparticles with controllable composition and structure and sometimes can be produced in large quantities [52,75]. All these properties influence the resulting antimicrobial activity and biocompatibility of nanoparticles, e.g., the biocompatibility of AgNPs might be easily enhanced by the reduction of their particle size. Furthermore, the surface modification (coating, functionalization, bioconjugation, etc.) of nanomaterials might serve to facilitate their applications in medicine [76].

The use of AgNPs (and also other silver nanostructures) in medicine as the antimicrobial coating of polymers has many advantages. The most important is that, in insignificant concentrations, silver is not toxic to human cells in normal use. It is also unlikely for a majority of microorganisms to develop resistance to silver, contrary to the usage of conventional and narrow-target antibiotics. This is due to silver attacking a broad range of targets in the bacterial cell (see Figure 6); in order to build resistance, bacterial cells would have to simultaneously develop a host of mutations to protect themselves. Thus, AgNPs have been used as antimicrobial coatings in various medical devices [77].

Samuel et al. [1] used uniform distributed AgNPs (10^12^–10^13^ of active nanoparticles per gram of polymer) in urinary catheters made from polyurethane and silicone. This use of nanosilver exhibited excellent antimicrobial activity against a broad spectrum of bacteria in vitro. They also observed a substantial reduction in catheter encrustation. Maneerung et al. [78] studied wound dressings made from bacterial cellulose. Bacterial cellulose is a very effective wound dressing because it can control wound exudates and provide a moist environment that is important for better wound healing. However, it exhibits no antimicrobial activity itself to prevent wound infection. Due to this, they impregnated this material with silver nanoparticles to achieve antimicrobial activity. They found that freeze-dried silver-nanoparticle-impregnated bacterial cellulose exhibited the strongest antimicrobial activity against Gram-negative *Escherichia coli* and Gram-positive *Staphylococcus aureus*, which are common bacteria found in contaminated wounds. Their wound dressing also lowered the possibility of damage to normal human tissue. Cohen et al. [79] coated a polypropylene surgical mesh with nanocrystalline silver nanoparticles. They studied the bactericidal effects of Ag-coated mesh against *S. aureus* and compared them with an uncoated one. They found that the coated mesh exhibited significant bactericidal activity. Due to this, nanocrystalline silver particles may decrease the incidence of postoperative prosthetic mesh infections. Loo et al. [80] developed silver nanoparticle–polyvinyl alcohol hydrogel coatings to reduce the incidence of bacterial colonization of endotracheal tubes. They incorporated silver nanoparticles into polyvinyl alcohol (PVA) to produce stable hydrogels. Their tests suggested that Ag-loaded PVA was nontoxic against human normal bronchial epithelial cells but effective against the attachment of *Pseudomonas aeruginosa* and *Staphylococcus aureus*, with a greater effect on *P. aeruginosa*.

#### 4.2.2. Silver Nanowires

As a representative of the group of one-dimensional nanostructures, silver nanowires were chosen to be described. Silver nanowires are an interesting object to study. Nowadays, they can be synthesized on a large scale and the formation of uniform nanowires of well-defined dimensions can be effectively controlled. Processing of silver into nanowires with well-defined dimensions may significantly enhance the performance and functionality of silver in most of its applications. The replacement of AgNPs by nanowires (with relatively higher aspect ratios) in polymer composites could greatly reduce the mechanical load of silver. Because bulk silver exhibits the highest electrical and thermal conductivity of all metals, silver nanowires have been extensively exploited in a variety of applications such as catalysis, electronics, photonics and medicine [81,82].

The excellent electrical and thermal conductivity of silver nanowires was successfully exploited in the area of thermal therapy. Thermal therapy is one of the most popular types of physiotherapy, primarily intended for the treatment of injured joints. It helps relieve pain, swelling, muscle weakness and numbness. In the past, the following materials were used for the thermal therapy: stretchable metal electrodes, such as long carbon nanotubes inserted into elastomers, and conductive elastomers based on AuNPs. Each of these materials has suitable properties for this application; however, it was necessary to develop a simple and inexpensive composite, which may be easily processed. Because of higher conductivity compared to C and a lower price than Au, the most suitable material for the thermal therapy is silver nanowires. One-dimensional silver nanowires also better maintain electrical conductivity during deformation (especially during stretching) than AgNPs [83]. Nowadays, applications of silver nanowires based on their excellent thermal conductivity are well established; however, their potential as promising antimicrobial coatings in medical devices is relatively untapped thus far. Moreover, the biocompatibility and antimicrobial activity of these materials can be easily enhanced.

**Figure 7:**
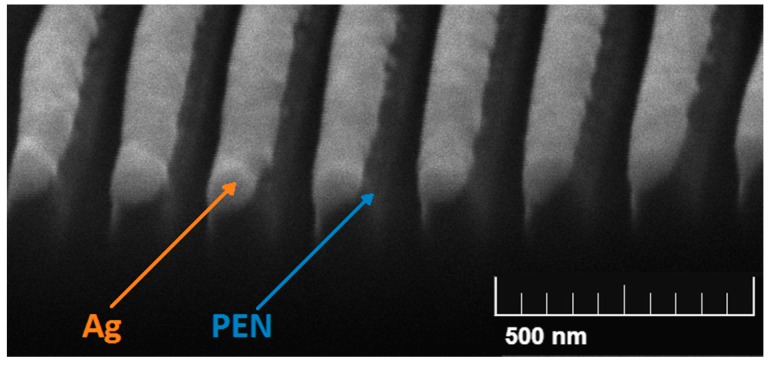
Scanning electron microscopy (SEM) image of silver nanowire arrays supported on polyethylenenaphthalate (PEN) [49].

Numerous studies have demonstrated the influence of surface morphology on material biocompatibility and antibacterial activity. The increase of specific surface area and roughness of antimicrobial coating using silver nanowire arrays, prepared by laser patterning and subsequent silver deposition (Figure 7), might be one effective way to do that [49]. The attachment and growth of human cells on artificial substrates can be affected by polymer surface modification, such as laser patterning. Thus, the surface modification enables direct control over the material biocompatibility, which might be significantly enhanced [84]. The surface roughness of biomaterials is one of the most important parameters that affects cell behaviour [85]. For example, the adhesion and proliferation of human vascular endothelial cells cultured on substrates with various surface roughnesses were studied by qualitative examinations of cell morphology and quantitative assessment of cell adhesion and proliferation rate. It was found that endothelial cell function was enhanced on the smooth solvent-cast surface rather than on the rough one [86]. Patterning has also been used to address the detailed molecular topology of focal adhesions. Cell adhesion and proliferation were promoted by a patterned model, in which molecularly well-defined adhesive spots were separated by non-adhesive regions [87]. The surface roughness also affected the resulting antibacterial effects of materials. It was found that augmenting microscale roughness caused an increase in bacterial accumulation; bacterial cells were easily anchored on the polymeric surface [88]. However, the influence of nanoscale surface roughness on bacterial colonization also had an opposite pattern, as in the study of Rimondini et al. [89], in which the nanoscale surface roughness played an important role in the reduction of plaque colonization on titanium. 

The significant antimicrobial potential of silver nanowires has been revealed in several studies, such as the study of Cui et al. [90], in which they found that the use of silver nanowires enhanced antibacterial properties and cell compatibility. They compared the antibacterial effects and cytocompatibility of untreated graphene oxide (GO) sheets with silver nanowire-treated ones. Their results suggested that the GO treated by Ag nanowires provided unprecedented antibacterial properties while maintaining the cell proliferation capacity necessary for enhancing the wide use of silver in medical applications. Tang et al. [91] prepared uniform and pure silver nanowires, which showed excellent and long-lasting antibacterial activity against *E. coli* and *S. aureus*, microorganisms that are frequently associated with nosocomial infections. Zhao et al. [92] developed a broad-spectrum and robust antimicrobial thin film coating based on large-area deposition of graphene-wrapped silver nanowires. They found that this hybrid coating showed broad-spectrum antimicrobial activity against Gram-negative bacteria *E. coli*, Gram-positive *S. aureus* and the fungus *Candida albicans*. The robust antimicrobial activity of the coating was reinforced by the encapsulation of Ag nanowires by graphene. The advantage of their antimicrobial coating consisted of its considerable transparency and flexibility.

As mentioned, the use of silver nanowires as antimicrobial coatings has tremendous advantages; however, in recent days, cytotoxic studies warning about the toxicity of silver nanostructures have begun to appear [53]. Some of these studies indicate that the most cytotoxic type of nanostructured silver materials are silver nanowires [92,93,94,95]. For this reason, the long-term applications of silver nanowires in healthcare are limited and their contact with living tissues should be minimized; these structures are potentially applicable only in vitro, e.g., integration into traumatic wound dressings and diabetic ulcers, treatment of dental and chirurgical instruments, etc. [49].

#### 4.2.3. Silver Nanolayers

Nanolayers are defined as thin layers with thicknesses ranging from 1 to 100 nm, hence belonging to the 2D nanostructures group. For many years, various types of thin films have been used to coat various types of materials, bringing them improved properties. Since the excellent antimicrobial properties of silver have been known from ancient times, the use of silver nanolayers as a surface treatment of polymeric materials was a logical choice. Such modification imparts to polymers exceptional antimicrobial properties [96]. 

**Figure 8:**
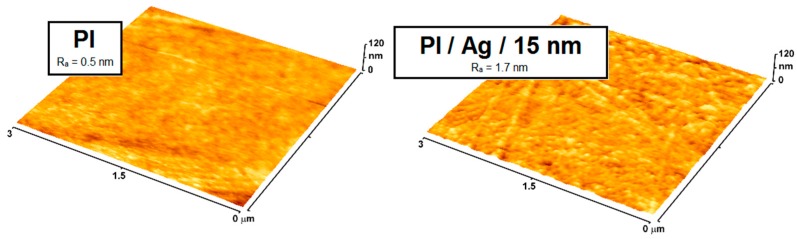
Atomic force microscopy (AFM) images of pristine polyimide (PI) and Ag nanolayer-coated PI, together with their surface roughnesses [48].

The development of a technology of thin silver layers has overcome the problem of the relatively high cost of metal silver, making possible the wide-spread use of this metal. Nowadays, nanoscale-thin silver layers covering less expensive materials use silver in a lower quantity while still benefiting from its antimicrobial effect; these have begun to be used widely in diverse areas, especially in the healthcare industry. The final result of such an approach led to extensive applications of Ag nanolayers to control hospital-acquired infections. Unlike untreated surfaces of polymeric medical devices, the high specific surface area of antimicrobial silver coatings and relatively increased nanoscale surface roughness are key elements of the enhanced functionality of medical devices such as dental implants, stents, hip prosthesis, and miscellaneous devices; in Figure 8 one can compare the surface morphology and roughness of Ag nanolayer-coated polyimide relative to an untreated one [48,97].

Due to the high specific surface area and relatively safe contact with human skin, silver nanolayers have been frequently used as antimicrobial coatings of dressing fibres to treat infected wounds, which relatively easily achieve the ion concentrations necessary to suppress infection. In addition, it is possible to supply a controlled dose of silver ions to the skin regions surrounding the wound, which accelerates wound healing and decreases the amounts of antibiotics required. Aleksandrova et al. [98] prepared a silver nanolayer on sintepon, one of the most widespread nonwoven polyester synthetics for the fabrication of dressings. They coated sintepon fibres with uniform Ag nanolayers of an average thickness of 40–50 nm and determined their antimicrobial activity using Gram-positive *Staphylococcus aureus* and Gram-negative *Escherichia coli* (both non-spore-forming bacteria), and the yeast *Candida albicans* (with a eukaryotic cell structure). They found that the Ag nanolayer coating caused a substantial increase in the antimicrobial and antiviral activity of this material and that synthetic fibres treated sparingly with soluble silver layers showed considerable promise as antimicrobial coatings. Dubas et al. [99] demonstrated that the layer-by-layer method can be effectively applied to prepare silver nanolayer coatings of silk or nylon fibres with the use of silver nanoparticles. Silk and nylon fibres were coated with layers of poly(diallyldimethylammonium chloride) and AgNPs capped with poly(methacrylic acid) (PDAMAC/PMAcapAg). The sequential dipping of nylon or silk fibres in dilute solutions led to the formation of a smooth and uniform thin film possessing excellent antimicrobial properties. Coated fibres were tested for their antimicrobial properties toward *Staphylococcus areus*. The results of antibacterial tests showed the percent inhibition of the bacteria as a function of the number of layers deposited. It is apparent that this technique could be used in the improvement of new synthetic or natural polymers where antimicrobial properties are required, such as polymeric medical devices associated with nosocomial infections. Carvalho et al. [100] modified the surface of polytetrafluoroethylene (PTFE) by the co-deposition of PTFE and polyamide (PA) thin films containing silver, while PA was used to attenuate the hydrophobic nature of PTFE. They tested these composites against hospital-isolated *P. aeruginosa* bacteria, which are able to degrade PTFE as a bulk material, as well as some of the developed thin films. They found that the polymer–metal combination induced the formation of a nanocomposite structure, which exhibited significant antimicrobial properties against *P. aeruginosa* without apparent degradation of the material.

**Figure 9:**
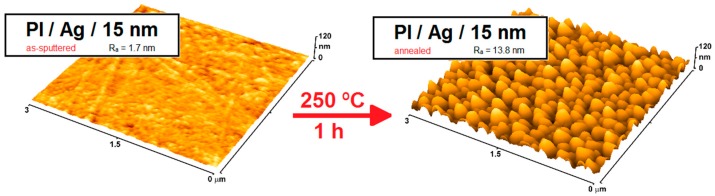
AFM images of silver-coated polyimide (PI) before (left) and after (right) annealing, together with their surface roughnesses [48].

As stated in the previous paragraph, thin silver films can be effectively used to enhance the functionality of polymeric materials. Nevertheless, one can efficiently use them to prepare structures with an increased surface area from nanolayers through a thermally induced transformation of thin metal layers into island-like structures. The formation of island-like structures with increased surface area might, in some cases, result in an enhanced antibacterial response. In several different studies, the formation of island-like structures after the annealing of thin layers was detected in the case of silver [14,48], palladium [51] and gold coatings [101]. Antibacterial properties of silver-coated polyimide, both before and after annealing, were examined using two model bacterial strains, *Escherichia coli* and *Staphylococcus epidermidis*, frequently involved in infections associated with a biofilm formation. Due to the structural transformation of a silver layer (see Figure 9), which increased the specific surface area of silver by an order of magnitude, the resulting antibacterial activity was significantly augmented. The larger the individual island-forming silver coating, the stronger the antibacterial effect observed. This effect diminishes once the individual silver islands in the transformed layer are interconnected [48]. 

In contrast, the thermal treatment of palladium-coated polyethylenenaphthalate, evaluated towards the same microorganisms, resulted in a completely opposite antibacterial effect. Detailed surface characterization uncovered the mechanism of transformation of the annealed surface: in response to annealing, Pd tends to aggregate and simultaneously forms a thin polymer layer, probably due to an injection of Pd into the soft underlying polymer. Thus, the changes in the surface morphology were induced by the synergetic effect of two processes: (i) the coalescence of Pd into separate nano-islands; and (ii) their partial embedding into the ultrathin surface polymer layer. Such Pd incorporation is, however, superficial. It could be linked to the superficial ultrathin (ones of nm) polymer overlay that almost reaches to the top of individual Pd islands. This phenomenon is known as a “curtain” effect. The submersion of Pd clusters into the polymer volume has a major impact on the resulting antibacterial activity. Even though the apparent specific surface area of Pd was increased due to the annealing process, no improvement in the resulting antibacterial properties was registered because of the “curtain” effect [51]. Obviously, the improvement of the antibacterial effects of metal nanolayers due to the increase in their surface area significantly depends on the specific combination of metal and polymer used.

### 4.3. Overview of Preparation Techniques of Silver Nanostructures 

Nowadays, nanotechnology is a widely developing area for the preparation of a variety of nanomaterials with unique properties. The synthesis of nanomaterials by a simple, low cost and high-yield technology has been a great challenge since the very early development of nanoscience. In addition, there is a growing need to develop eco-friendly processes that do not use toxic chemicals in the synthesis protocols. Plenty of methods might be used for their successful preparation. All these preparation techniques can be divided into two methods based on quite opposite approaches: (i) top-down processes, which start with the bulk material and transform it to the nanoscale; and (ii) bottom-up processes, which start with the individual atoms and build them upwards to the nanostructured form [102,103]. Recently, combinations of both have also been used [104]. Examples of bottom-up and top-down techniques are listed in Table 4.

#### 4.3.1. Silver Nanoparticles

Silver nanoparticles have been studied owing to their unique properties, such as size- and shape-dependent optical, antimicrobial, and electrical properties. As mentioned, their preparation techniques include top-down processes, in which the particles are made smaller from bulk metal, and bottom-up processes, where the individual atoms of NPs are formed from the solution and their sizes are precisely controlled. The preparation techniques of AgNPs can be divided into three groups based on their mechanism. These are physical, chemical and biological methods.

Physical methods have many advantages in comparison with chemical ones. They excel due to the absence of solvent contamination in the final products and prepared NPs being uniformly distributed. Physical methods are simple and relatively fast. The prepared nanoparticles can have a variety of sizes, shapes and chemical natures. It is also possible to prepare small nanoparticles in high concentrations, and the particle generation is very stable. Some types of physical techniques are enumerated in Table 5 [105,106].

Chemical methods are based on (i) reduction by organic and/or inorganic reducing agents; or (ii) precipitation of metals, in the presence of stabilizing agents suitable for corresponding metal atoms. In general, various reducing agents, such as sodium citrate, ascorbate, borohydride, elemental hydrogen, etc., have already been used for the reduction of silver ions in aqueous or non-aqueous solutions. First, precipitated atoms became centres of nucleation, which in turn leads to the formation of atomic clusters. The clusters are surrounded by stabilizing molecules (e.g., thiols, amines, acids, and alcohols), which stabilize (terminate) particle growth and protect particles from sedimentation, agglomeration, or losing their surface properties. The advantages of chemical methods are the reproducibility, availability of reactants and low cost. However, these methods require a long time and special experimental conditions. Several examples of chemical-based preparation methods are shown in Table 5 [105,107].

Numerous studies suggest that, due to the use of toxic chemicals, chemical methods of nanoparticle synthesis are non-ecological and the use of hazardous chemicals significantly limits their biomedical applications. For this reason, the green synthesis of NPs by biological methods has been widely developed (Table 5). The potential organisms for the synthesis of nanoparticles range from simple prokaryotic to complex eukaryotic cells. For example, microorganisms including bacteria, fungi, algae, yeast and actinomycetes have already been used. Also, the usage of extracts from various plants, i.e., diverse gymnosperms and angiosperms, is possible. By these green methods, highly stable and well-characterized NPs can be prepared effectively. The size and morphology of NPs can be effectively controlled by an adjustment of several parameters, such as concentration of substrate, pH, light, temperature, buffer strength, electron donor (e.g., glucose or fructose), biomass and substrate concentration, mixing speed and exposure time [105,106,107,108].

#### 4.3.2. Silver Nanowires

Nanowires are predominately defined by the geometry of their cross sections; one can observe the nanowire formation and the mechanisms by which they attain a state of minimum free energy. Similar to other nanostructures, nanowires can be prepared by top-down and bottom-up approaches. Top-down methods involve the extraction of nanowire from a bulk material by processes such as electron-beam lithography or mechanical reduction, while bottom-up ones include the growth of nanowires by chemical or molecular assembly or template-assisted electrodeposition. All of these methods can also be classified as physical or chemical methods; however, for this type of nanostructure, division into solution-based and gas-phase methods is the most revealing (see Table 6) [109]. 

Solution-phase methods usually require the presence of surfactants, which form micelles or inverse micelles. In addition, the use of templates, in combination with appropriate chemical or electrochemical reactions, is often required for the successful preparation of nanowires in this way. However, these techniques are especially appealing because of the low-growth temperatures, potential for scaling-up, and capability of producing high-density arrays. There is also the possibility to prepare ultrathin nanowires with a high aspect ratio and excellent stability. Due to these advantages, such nanowires could be assembled into functional devices in many areas including antimicrobials [110].

Gas-phase methods are batch processes. The precursor vapour, necessary for these processes, is often generated at first. It is transported from a high-temperature zone to a low-temperature zone. Then, part of the vapour diffuses and is deposited on the substrate, where nanowires nucleate to grow. The advantages of gas-phase syntheses are also numerous. Generally, these techniques provide a much cleaner product than solution-based ones. The ability to exactly manage the process parameters allows for precise control of the shape, size and chemical composition of the nanowires formed. The large-scale industrial application of these techniques has been successfully commercialized after considerable progress in the development of new products [111].

In light of all these preparation methods, vapour–liquid–solid growth (VLS) is the most frequently used method for the preparation of nanowires. For this reason, its mechanism will be shortly discussed. The VLS method runs as a 1D crystal growth assisted by a metal catalyst, which results in the formation of whiskers, rods and wires. At a high temperature, the catalyst forms liquid alloy drips by the absorption of vapour components. Then, the alloy became supersaturated. Thereafter, the precipitation of the component at the liquid–solid interface occurs to achieve the minimum free energy of the alloy system. This begins the 1D crystal growth, which continues as long as the vapour components are supplied. Because of the presence of vapour (carrier of solid components), liquid (catalyst of alloy) and solid (precipitated one-dimensional structures) phases, the mechanism is called VLS [112].

#### 4.3.3. Silver Nanolayers

Nanolayers, thin layers of nanoscale thickness, can be prepared by various techniques. Like other types of nanostructures, the most common classification of their preparation techniques is into chemical and physical methods (Table 7). Physical methods (i.e., evaporation and sputtering) include deposition techniques based on evaporating or expulsion of the material from its source. Chemical methods work on the basis of a specific chemical reaction. This reaction is then based, for example, on the electrical separation of ions (electroplating) or on the thermal effects and increasing temperature during the deposition from the vapour (thermal chemical vapour deposition) [113]. 

The motivation for the use of physical methods lies in the specific characteristics of the process and deposited layers. A wide range of thicknesses from tens of nanometers to tens of micrometers, with high uniformity and reproducibility, can be effectively achieved. There are no limitations in the selection of the source of initial material; metals, semiconductors, glass, ceramics and plastics can be transformed into a thin layer during physical processes. The multi-layer systems may be formed by one process, and a low surface temperature can be maintained. The bottom layer is not destroyed. The properties of the layers, such as their resistivity, temperature coefficient of resistance (TCR), adhesion, structure, composition, density and refractive index, may be enhanced by adjusting the process parameters (e.g., substrate temperature, process pressure, energy, deposition rate, etc.) [114,115].

Chemical methods for the preparation of nanolayers provide highly pure materials ay the atomic or nanometer scale. Single or multilayers, composites and functionally graded nanostructured films with controllable size and unique structure can be formed by these techniques. The versatility of chemical methods led to the rapid spread of their use, and for this reason they became one of the main methods for the deposition of thin films for a wide range of applications [113,116,117].

## 5. Conclusions 

Over the past few decades, the use of polymeric medical devices in healthcare applications has been extended considerably. However, their long-term applications were often associated with biofilm formation, which leads to the development of nosocomial infections. This paper discusses those problems. The mechanism of the biofilm formation was explained. Then, the methods of the antimicrobial treatment of polymeric medical devices were briefly described, including antibiotics, antiseptics, non-antibiotics and noble metals. Because silver is the gold standard in nanoscale antimicrobial treatment, the last part of the review focused on diverse forms of nanostructured-silver-treated polymers. The mechanism of action of silver nanostructures was summarized. Multifaceted mechanisms of action of Ag^+^ ions prevent the development of bacterial resistance, and, for this reason, nanostructured silver is an excellent candidate for the preparation of antimicrobial coatings of polymeric medical devices. The applications of three types of silver nanostructures (nanoparticles, nanowires, and nanolayers) in medicine were reviewed. Their medical applications in the literature are considerable; they relate to various types of polymeric medical devices, such as catheters, endotracheal tubes, wound dressings, surgical mesh and other polymeric fibres. Nowadays, there are many techniques for the preparation of silver and other noble metal nanostructures. Nevertheless, for the preparation of nanostructured antimicrobial coatings of polymers, methods focusing on direct nanostructure formation on the polymeric substrate are most suitable. Otherwise, after the preparation of nanostructures, the next step, based on their incorporation into polymeric matrix, requires further research. 

## Figures and Tables

**Table 1:** Types of polymeric medical devices associated with nosocomial infections.

Microbial Species	Medical Devices	Examples
*Staphylococci*, *Enterobacteriaceae*, *Enterococci* and *Candida species*	Catheters	Blood vessel catheter, CAPD catheters
	Tubes	Cerebrospinal fluid shunts, endotracheal tubes
	Cardiological implants	Arterial grafts, cardiac valves, pacemaker electrodes, total artificial hearts
	Prostheses	Total joint replacements (endoprostheses), ocular and penile prostheses
*Enterobacteriaceae* and *Enterococci*	Urinary catheters	Transurethral, suprapubic, and nephrostomy catheters,
	Urinary stents	Double-J stents

CAPD: continuous ambulatory peritoneal dialysis [16,17,18].

**Table 2:** Methods of antimicrobial treatment of polymeric medical devices.

Type of Treatment	Treatment Method	Example
Antibiotics	Coating	Mynocycline, rifampin [28]
	Covalent bond	Vancomycin [29]
	Ionic bond	Silver sulfadiazine [30]
	Matrix	Ceftazidime and gentamicin implants [31]
Antiseptics	Impregnation	Chlorhexidine [32]
Non-antibiotics	Coating Synergism with antibiotics	Methylene blue and its derivatives [33] Phenothiazine and its derivatives [34]
Noble metals	Incorporation	Silver salts, ions and complexes [35,36,37]
	Incorporation, Coating	Nanostructured silver, gold, copper and palladium [12,38,39,40]

**Table 3:** Medical applications of nanosilver [43,44,52,53].

Prevention	Therapy
Bone cements	Wound dressings
Neurosurgical shunts	Creams and ointments
Venous catheters	Epistaxis
Cardiovascular implants	Gonococcal eye infection
Contact lenses	Pleurodesis
Dental and surgical instruments	Granulomas

**Table 4:** Examples of bottom-up and top-down approaches [102,103,104].

Bottom-Up	Top-Down
Supercritical fluid synthesis	Mechanical milling
Spinning	Etching
Chemical vapour deposition	Electro-explosion
Plasma spraying and flame spraying	Sputtering
Molecular beam epitaxy	Laser ablation
Sol and Sol-Gel process	Dry grinding system
Laser pyrolysis	Wet grinding system
Aerosol based process	Ultrasonic wave
Atomic or molecular condensation	Mechanical alloying method
Using of templates	Lithography
Chemical reduction	Cutting

**Table 5:** The overview of the preparation techniques of AgNPs [105,106,107,108].

Physical	Chemical	Biological
Laser ablation	Electro-explosion	Microorganism
Irradiation	Sol-gel synthesis	Bio-templates
Pulse vapour deposition	Chemical reduction	Plant-extract-assisted biogenesis
Ultrasonication	Microemulsion method	
Microwave method	Hydrothermal synthesis	
Electro chemical	Polyol synthesis	
Thermal evaporation	Microwave-assisted synthesis	
Lithography	Indirect reduction	
Melt mixing	UV-initiated photoreduction	
Electrospraying	.Photoinduced reduction	
Inert gas condensation	Electrochemical synthesis	

**Table 6:** The outline into the preparation methods of silver nanowires [109,110,111,112].

Solution-Based	Gas-Phase
Solution–liquid–solid method	Vapour–liquid–solid growth
Solvothermal method	Physical vapour deposition
Template-based method	Chemical vapour deposition
Fluid–liquid–solid method	Vapour–solid growth
Sol-gel synthesis	Oxide-assisted growth
Chemical bath deposition	Laser ablation
Electrochemical deposition	Aerotaxy-based growth
Hydrothermal method	Electrodeless etching
Aqueous chemical growth	Thermal evaporation
Electrospraying	Carbothermal reduction
Hot-injection method	Flame-based synthesis
Microemulsion	Lithography

**Table 7:** The division of deposition techniques for the preparation of silver nanolayers [113,114,115,116].

Physical		Chemical	
Evaporation	Ion plating, laser ablation, molecular beam exipaxy, electron beam, thermal and vacuum evaporation	Plating Sol-Gel	Electroplating Electrolysis
Sputtering	Ion beam, reactive, magnetron, high target utilization, ion-assisted and gas flow sputtering	Chemical vapour deposition	Metalorganic vapour phase epitaxy, plasma-enhanced and thermal deposition

## References

[1] Samuel U., Guggenbichlerb J.P. (2004). Prevention of catheter-related infections: The potential of a new nano-silver impregnated catheter. Int. J. Antimicrob. Agents.

[2] Mangram A.J., Horan T.C., Pearson M.L., Silver L.C., Jarvis W.R. (1999). Guideline for prevention of surgical site infection, 1999. Am. J. Infect. Control.

[3] Richards M.J., Edwards J.R., Culver D.H., Gaynes R.P. (2000). Nosocomial infections in combined medical-surgical intensive care units in the United States. Infect. Control Hosp. Epidemiol..

[4] Maki D.G., Cobb L., Garman J.K., Shapiro J.M., Ringer M., Helgerson R.B. (1988). An attachable silver-impregnated cuff for prevention of infection with central venous catheters: A prospective randomized multicenter trial. Am. J. Med..

[5] Weber D.J., Raasch R., Rutala W.A. (1999). Nosocomial Infections in the ICU: The Growing Importance of Antibiotic-Resistant Pathogens. Chest.

[6] Weinstein R.A. (1998). Nosocomial infection update. Emerg. Infect. Dis..

[7] Munoz-Bonilla A., Fernandez-Garcia M. (2012). Polymeric materials with antimicrobial activity. Prog. Polym. Sci..

[8] Timofeeva L., Kleshcheva N. (2011). Antimicrobial polymers: Mechanism of action, factors of activity, and applications. Appl. Microbiol. Biotechnol..

[9] Tashiro T. (2001). Antibacterial and bacterium adsorbing macromolecules. Macromol. Mater. Eng..

[10] Gabriel G.J., Som A., Madkour A.E., Eren T., Tew G.N. (2007). Infectious disease: Connecting innate immunity to biocidal polymers. Mater. Sci. Eng. R..

[11] Page K., Wilson M., Parkin I.P. (2009). Antimicrobial surfaces and their potential in reducing the role of the inanimate environment in the incidence of hospital-acquired infections. J. Mater. Chem..

[12] Roe D., Karandikar B., Bonn-Savage N., Gibbins B., Roullet J.B. (2008). Antimicrobial surface functionalization of plastic catheters by silver nanoparticles. J. Antimicrob. Chemother..

[13] Yao Y., Ohko Y., Sekiguchi Y., Fujishima A., Kubota Y. (2008). Self-sterilization using silicone catheters coated with Ag and TiO_2_ nanocomposite thin film. J. Biomed. Mater. Res. Part B Appl. Biomater..

[14] Siegel J., Polivkova M., Kasalkova N.S., Kolska Z., Svorcik V. (2013). Properties of silver nanostructure-coated PTFE and its biocompatibility. Nanoscale Res. Lett..

[15] Tamboli M.S., Kulkarni M.V., Patil R.H., Gade W.N., Navale S.C., Kale B.B. (2012). Nanowires of silver–polyaniline nanocomposite synthesized via in situ polymerization and its novel functionality as an antibacterial agent. Colloids Surf. B Biointerfaces.

[16] Feldman C., Kassel M., Cantrell J., Kaka S., Morar R., Mahomed A.G., Philips J.I. (1999). The presence and sequence of endotracheal tube colonization in patients undergoing mechanical ventilation. Eur. Respir. J..

[17] Pfaller M.A. (1996). Nosocomial Candidiasis: Emerging Species, Reservoirs, and Modes of Transmission. Clin. Infect. Dis..

[18] Schierholz J.M., Beuth J. (2001). Implant infections: A haven for opportunistic bacteria. J. Hosp. Infect..

[19] Flemming H.C. (1998). Relevance of biofilms for the biodeterioration of surfaces of polymeric materials. Polym. Degrad. Stable.

[20] Gao G.Z., Lange D., Hilpert K., Kindrachuk J., Zou Y.Q., Cheng J.T.J., Kazemzadeh-Narbat M., Yu K., Wang R.Z., Straus S.K. (2011). The biocompatibility and biofilm resistance of implant coatings based on hydrophilic polymer brushes conjugated with antimicrobial peptides. Biomaterials.

[21] Bergogne-Bérézin E., Decreé D., Joly-Guillou M.L. (1993). Opportunistic nosocomial multiply resistant bacterial infections—their treatment and prevention. J. Antimicrob. Chemother. A.

[22] Handwerger S., Raucher B., Altarac D., Monka J., Marchione S., Singh K.V., Murray B.E., Wolff J., Walters B. (1993). Nosocomial outbreak due to *Enterococcus faecium* highly resistant to vancomycin, penicillin, and gentamicin. Clin. Infect. Dis..

[23] Clark N.C., Hill B.C., O’Hara C.M., Steingrimsson O., Cooksey R.C. (1990). Epidemiologic typing of *Enterobacter sakazakii* in two neonatal nosocomial outbreaks. Diagn. Microbiol. Infect. Dis..

[24] Jarvis W.R. (1995). Epidemiology of nosocomial fungal infections, with emphasis on *Candida species*. Clin. Infect. Dis..

[25] Wong G.K.C., Ip M., Poon W.S., Mak C.W.K., Ng R.Y.T. (2010). Antibiotics-impregnated ventricular catheter versus systemic antibiotics for prevention of nosocomial CSF and non-CSF infections: A prospective randomised clinical trial. J. Neurol. Neurosurg. Psychiatry.

[26] Kristiansen J.E., Amaral L. (1997). The potential management of resistant infections with non-antibiotics. J. Antimicrob. Chemother..

[27] Toracchio S., Marzio L. (2003). Primary and secondary antibiotic resistance of *Helicobacter pylori* strains isolated in central Italy during the years 1998–2002. Dig. Liver Dis..

[28] Chemaly R.F., Sharma P.S., Youssef S., Gerber D., Hwu P., Hanmod S.S., Jiang Y., Hachem R.Y., Raad I.I. (2010). The efficacy of catheters coated with minocycline and rifampin in the prevention of catheter-related bacteremia in cancer patients receiving high-dose interleukin-2. Int. J. Infect. Dis..

[29] Jose B., Antoci V., Zeiger A.R., Wickstrom E., Hickok N.J. (2005). Vancomycin covalently bonded to titanium beads kills *Staphylococcus aureus*. Chem. Biol..

[30] Fox C.L., Modak S.M. (1974). Mechanism of silver sulfadiazine action on burn wound infections. Antimicrob. Agents Chemother..

[31] Elsner J.J., Berdicevsky I., Zilberman M. (2011). In vitro microbial inhibition and cellular response to novel biodegradable composite wound dressings with controlled release of antibiotics. Acta Biomater..

[32] Sheng W.H., Wang J.T., Chang S.C., Hsueh P.R., Luh K.T. (2000). Evaluation of antiseptic-impregnated central venous catheters for prevention of catheter-related infection in intensive care unit patients. Diagn. Microbiol. Infect. Dis..

[33] Wainwright M., Phoenix D.A., Gaskell M., Marshall B. (1999). Photobactericidal activity of methylene blue derivatives against vancomycin-resistant *Enterococcus spp*.. J. Antimicrob. Chemother..

[34] Rahbar M., Mehrgan H., Hadji-Nejad S. (2010). Enhancement of Vancomycin Activity by phenothiazines against vancomycin-resistant *Enterococcus faecium* in vitro. Basic Clin. Pharmacol. Toxicol..

[35] Balazs D.J., Triandafillu K., Wood P., Chevolot Y., van Delden C., Harms H., Hollenstein C., Mathieu H.J. (2004). Inhibition of bacterial adhesion on PVC endotracheal tubes by RF-oxygen glow discharge, sodium hydroxide and silver nitrate treatments. Biomaterials.

[36] Becker R.O., Spadaro J.A. (1978). Treatment of orthopaedic infections with electrically generated silver ions: A preliminary report. J. Bone Jt. Surg. Am..

[37] Panzner M.J., Deeraksa A., Smith A., Wright B.D., Hindi K.M., Kascatan-Nebioglu A., Torres A.G., Judy B.M., Hovis C.E., Hilliard J.K. (2009). Synthesis and in vitro efficacy studies of silver carbene complexes on biosafety level 3 bacteria. Eur. J. Inorg. Chem..

[38] Li X.N., Robinson S.M., Gupta A., Saha K., Jiang Z.W., Moyano D.F., Sahar A., Riley M.A., Rotello V.M. (2014). Functional gold nanoparticles as potent antimicrobial agents against multi-drug-resistant bacteria. ACS Nano.

[39] Ben-Sasson M., Zodrow K.R., Qi G.G., Kang Y., Giannelis E.P., Elimelech M. (2014). Surface functionalization of thin-film composite membranes with copper nanoparticles for antimicrobial surface properties. Environ. Sci. Technol..

[40] Adams C.P., Walker K.A., Obare S.O., Docherty K.M. (2014). Size-dependent antimicrobial effects of novel palladium nanoparticles. PLoS ONE.

[41] Maki D.G., Stolz S.M., Wheeler S., Mermel L.A. (1997). Prevention of central venous catheter-related bloodstream infection by use of an antiseptic-impregnated catheter: A randomized, controlled trial. Ann. Intern. Med..

[42] Martins M., Dastidar S.G., Fanning S., Kristiansen J.E., Molnar J., Pages J.M., Schelz Z., Spengler G., Viveiros M., Amaral L. (2008). Potential role of non-antibiotics (helper compounds) in the treatment of multidrug-resistant Gram-negative infections: Mechanisms for their direct and indirect activities. Int. J. Antimicrob. Agents.

[43] Lansdown A.B. (2006). Silver in health care: Antimicrobial effects and safety in use. Curr. Probl. Dermatol..

[44] Mijnendonckx K., Leys N., Mahillon J., Silver S., van Houdt R. (2013). Antimicrobial silver: Uses, toxicity and potential for resistance. Biomaterials.

[45] Aflori M., Miron C., Dobromir M., Drobota M. (2015). Bactericidal effect on Foley catheters obtained by plasma and silver nitrate treatments. High Perform. Polym..

[46] Kascatan-Nebioglu A., Panzner M.J., Tessier C.A., Cannon C.L., Youngs W.J. (2007). N-Heterocyclic carbene-silver complexes: A new class of antibiotics. Coord. Chem. Rev..

[47] Siegel J., Staszek M., Polivkova M., Reznickova A., Rimpelova S., Svorcik V. (2016). Green synthesized noble metals for biological applications. Mater. Today Proc..

[48] Siegel J., Polivkova M., Staszek M., Kolarova K., Rimpelova S., Svorcik V. (2015). Nanostructured silver coatings on polyimide and their antibacterial response. Mater. Lett..

[49] Polivkova M., Štrublová V., Hubáček T., Rimpelová S., Švorčík V., Siegel J. (2016). Surface characterization and antibacterial response of silver nanowire arrays supported on laser-treated polyethylene naphthalate. Mater. Sci. Eng. C.

[50] Sotiriou G.A., Pratsinis S.E. (2011). Engineering nanosilver as an antibacterial, biosensor and bioimaging material. Curr. Opin. Chem. Eng..

[51] Polivkova M., Valova M., Siegel J., Rimpelova S., Hubacek T., Lyutakov O., Svorcik V. (2015). Antibacterial properties of palladium nanostructures sputtered on polyethylene naphthalate. RSC Adv..

[52] Chaloupka K., Malam Y., Seifalian A.M. (2010). Nanosilver as a new generation of nanoproduct in biomedical applications. Trends Biotechnol..

[53] Chen X., Schluesener H.J. (2008). Nanosilver: A nanoproduct in medical application. Toxicol. Lett..

[54] Yamanaka M., Hara K., Kudo J. (2005). Bactericidal actions of a silver ion solution on Escherichia coli, studied by energy-filtering transmission electron microscopy and proteomic analysis. Appl. Environ. Microbiol..

[55] Yoshida K., Tanagawa M., Atsuta M. (1999). Characterization and inhibitory effect of antibacterial dental resin composites incorporating silver-supported materials. J. Biomed. Mater. Res. A.

[56] Kittler S., Greulich C., Diendorf J., Koller M., Epple M. (2010). Toxicity of silver nanoparticles increases during storage because of slow dissolution under release of silver ions. Chem. Mater..

[57] Chernousova S., Epple M. (2013). Silver as antibacterial agent: Ion, nanoparticle, and metal. Angew. Chem. Int. Ed..

[58] Jung W.K., Koo H.C., Kim K.W., Shin S., Kim S.H., Park Y.H. (2008). Antibacterial activity and mechanism of action of the silver ion in *Staphylococcus aureus* and *Escherichia coli*. Appl. Environ. Microb..

[59] Schierholz J.M., Wachol-Drewek Z., Lucas L.J., Pulverer G. (1998). Activity of silver ions in different media. Zent. Bl. Bakteriol..

[60] Jansen B., Rinck M., Wolbring P., Strohmeier A., Jahns T. (1994). In vitro evaluation of the antimicrobial efficacy and biocompatibility of a silver-coated central venous catheter. J. Biomater. Appl..

[61] Osińska-Jaroszuk M., Ginalska G., Belcarz A., Uryniak A. (2009). Vascular prostheses with covalently bound gentamicin and amikacin reveal superior antibacterial properties than silver-impregnated ones: An in vitro study. Eur. J. Vasc. Endovasc. Surg..

[62] Guggenbichler J.P., Boswald M., Lugauer S., Krall T. (1999). A new technology of microdispersed silver in polyurethane induces antimicrobial activity in central venous catheters. Infection.

[63] Trooskin S.Z., Donetz A.P., Baxter J., Harvey R.A., Greco R.S. (1989). Infection-resistant continuous peritoneal dialysis catheters. Nephron.

[64] Jansen B., Jansen S., Peters G., Pulverer G. (1992). In Vitro efficacy of a central venous catheter (‘Hydrocath’) loaded with teicoplanin to prevent bacterial colonization. J. Hosp. Infect..

[65] Raad I., Darouiche R., Hachem R., Mansouri M., Bodey G.P. (1996). The broad-spectrum activity and efficacy of catheters coated with minocycline and rifampin. J. Infect. Dis..

[66] Hampl J., Schierholz J., Jansen B., Aschoff A. (1995). In vitro and in vivo efficacy of a rifampin-loaded silicone catheter for the prevention of CSF shunt infections. Acta Neurochir..

[67] Groeger J.S., Lucas A.B., Coit D., Laquaglia M., Brown A.E., Turnbull A., Exelby P. (1993). A prospective, randomized evaluation of the effect of silver impregnated subcutaneous cuffs for preventing tunneled chronic venous access catheter infections in cancer patients. Ann. Surg..

[68] Bassetti S., Hu J., d’Agostino R.B., Sherertz R.J. (2001). Prolonged antimicrobial activity of a catheter containing chlorhexidine-silver sulfadiazine extends protection against catheter infections in vivo. Antimicrob. Agents Chemother..

[69] Braydich-Stolle L., Hussain S., Schlager J.J., Hofmann M.C. (2005). In vitro cytotoxicity of nanoparticles in mammalian germline stem cells. Toxicol. Sci..

[70] Diakowska D., Lewandowski A., Kopec W., Diakowski W., Chrzanowska T. (2007). Oxidative DNA damage and total antioxidant status in serum of patients with esophageal squamous cell carcinoma. Hepatogastroenterology.

[71] Ahamed M., Karns M., Goodson M., Rowe J., Hussain S.M., Schlager J.J., Hong Y.L. (2008). DNA damage response to different surface chemistry of silver nanoparticles in mammalian cells. Toxicol. Appl. Pharmacol..

[72] Huang Y., Duan X.F., Wei Q.Q., Lieber C.M. (2001). Directed assembly of one-dimensional nanostructures into functional networks. Science.

[73] Xia Y.N., Yang P.D., Sun Y.G., Wu Y.Y., Mayers B., Gates B., Yin Y.D., Kim F., Yan Y.Q. (2003). One-dimensional nanostructures: Synthesis, characterization, and applications. Adv. Mater..

[74] Staszek M., Siegel J., Rimpelova S., Lyutakov O., Svorcik V. (2015). Cytotoxicity of noble metal nanoparticles sputtered into glycerol. Mater. Lett..

[75] Sun Y.G., Xia Y.N. (2002). Shape-controlled synthesis of gold and silver nanoparticles. Science.

[76] Kim J.S., Kuk E., Yu K.N., Kim J.H., Park S.J., Lee H.J., Kim S.H., Park Y.K., Park Y.H., Hwang C.Y. (2014). Antimicrobial effects of silver nanoparticles. Nanomedicine.

[77] Pal S., Tak Y.K., Song J.M. (2007). Does the antibacterial activity of silver nanoparticles depend on the shape of the nanoparticle? A study of the gram-negative bacterium Escherichia coli. Appl. Environ. Microbiol..

[78] Maneerung T., Tokura S., Rujiravanit R. (2008). Impregnation of silver nanoparticles into bacterial cellulose for antimicrobial wound dressing. Carbohydr. Polym..

[79] Cohen M.S., Stern J.M., Vanni A.J., Kelley R.S., Baumgart E., Field D., Libertino J.A., Summerhayes I.C. (2007). In vitro analysis of a nanocrystalline silver-coated surgical mesh. Surg. Infect..

[80] Loo C.Y., Young P.M., Lee W.H., Cavaliere R., Whitchurch C.B., Rohanizadeh R. (2014). Non-cytotoxic silver nanoparticle-polyvinyl alcohol hydrogels with anti-biofilm activity: Designed as coatings for endotracheal tube materials. Biofouling.

[81] Sun Y.G., Gates B., Mayers B., Xia Y.N. (2002). Crystalline silver nanowires by soft solution processing. Nano Lett..

[82] Sun Y.G., Xia Y.N. (2002). Large-scale synthesis of uniform silver nanowires through a soft, self-seeding, polyol process. Adv. Mater..

[83] Choi S., Park J., Hyun W., Kim J., Kim J., Lee Y.B., Song C., Hwang H.J., Kim J.H., Hyeon T. (2015). Stretchable heater using ligand-exchanged silver nanowire nanocomposite for wearable articular thermotherapy. ACS Nano.

[84] Rebollar E., Frischauf I., Olbrich M., Peterbauer T., Hering S., Preiner J., Hinterdorfer P., Romanin C., Heitz J. (2008). Proliferation of aligned mammalian cells on laser-nanostructured polystyrene. Biomaterials.

[85] Mirzadeh H., Dadsetan M. (2003). Influence of laser surface modifying of polyethylene terephthalate on fibroblast cell adhesion. Radiat. Phys. Chem..

[86] Xu C.Y., Yang F., Wang S., Ramakrishna S. (2004). In vitro study of human vascular endothelial cell function on materials with various surface roughness. J. Biomed. Mater. Res..

[87] Arnold M., Cavalcanti-Adam E.A., Glass R., Blümmel J., Eck W., Kantlehner M., Kessler H., Spatz J.P. (2004). Activation of integrin function by nanopatterned adhesive interfaces. Chemphyschem..

[88] Bollen C.M.L., Lambrechts P., Quirynen M. (1997). Comparison of surface roughness of oral hard materials to the threshold surface roughness for bacterial plaque retention: A review of the literature. Dent. Mater..

[89] Rimondini L., Faré S., Brambilla E., Felloni A., Consonni C., Brossa F., Carrassi A. (1997). The effect of surface roughness on early in vivo plaque colonization on titanium. J. Periodontol..

[90] Cui J.H., Liu Y.L. (2015). Preparation of graphene oxide with silver nanowires to enhance antibacterial properties and cell compatibility. RSC Adv..

[91] Tang C.L., Sun W., Lu J.M., Yan W. (2014). Role of the anions in the hydrothermally formed silver nanowires and their antibacterial property. J. Colloid Interface Sci..

[92] Zhao C., Deng B., Chen G.C., Lei B., Hua H., Peng H.L., Yan Z.M. (2016). Large-area chemical vapor deposition-grown monolayer graphene-wrapped silver nanowires for broad-spectrum and robust antimicrobial coating. Nano Res..

[93] Stoehr L.C., Gonzalez E., Stampfl A., Casals E., Duschl A., Puntes V., Oostingh G. J. (2011). Shape matters: Effects of silver nanospheres and wires on human alveolar epithelial cells. Part. Fibre Toxicol..

[94] Zhang T., Wang L., Chen Q., Chen C. (2014). Cytotoxic potential of silver nanoparticles. Yonsei Med. J..

[95] Kim M. J., Shin S. (2014). Toxic effects of silver nanoparticles and nanowires on erythrocyte rheology. Food Chem. Toxicol..

[96] Siegel J., Jurik P., Kolska Z., Svorcik V. (2013). Annealing of silver nanolayers sputtered on polytetrafluoroethylene. Surf. Interface Anal..

[97] Chinnasamy R., Krishnamoorthy R., Shamugam R.K., Thangavelu R.R. (2013). Synthesis and antibacterial studies of nanostructured Ag thin films. Adv. Mater. Res..

[98] Aleksandrova T.P., Vais A.A., Masliy A.I., Burmistrov V.A., Gusev A.A., Bagavieva S.K. (2015). Synthetic fibers with silver-containing coatings and their antimicrobial properties. Mater. Manuf. Process.

[99] Dubas S.T., Kumlangdudsana P., Potiyaraj P. (2006). Layer-by-layer deposition of antimicrobial silver nanoparticles on textile fibers. Colloids Surf. A.

[100] Carvalho D., Sousa T., Morais P.V., Piedade A.P. (2016). Polymer/metal nanocomposite coating with antimicrobial activity against hospital isolated pathogen. Appl. Surf. Sci..

[101] Siegel J., Krajcar R., Kolska Z., Hnatowicz V., Svorcik V. (2011). Annealing of gold nanostructures sputtered on polytetrafluoroethylene. Nanoscale Res. Lett..

[102] Wang Y.L., Xia Y.N. (2004). Bottom-up and top-down approaches to the synthesis of monodispersed spherical colloids of low melting-point metals. Nano Lett..

[103] Biswas A., Bayer I.S., Biris A.S., Wang T., Dervishi E., Faupel F. (2012). Advances in top-down and bottom-up surface nanofabrication: Techniques, applications & future prospects. Adv. Colloid Interface Sci..

[104] Xu C.A., van Zalinge H., Pearson J.L., Glidle A., Cooper J.M., Cumming D.R.S., Haiss W., Yao J.L., Schiffrin D.J., Proupin-Perez M. (2006). A combined top-down bottom-up approach for introducing nanoparticle networks into nanoelectrode gaps. Nanotechnology.

[105] Iravani S., Korbekandi H., Mirmohammadi S.V., Zolfaghari B. (2014). Synthesis of silver nanoparticles: Chemical, physical and biological methods. Res. Pharm. Sci..

[106] Gudikandula K., Maringanti S.C. (2016). Synthesis of silver nanoparticles by chemical and biological methods and their antimicrobial properties. J. Exp. Nanosci..

[107] Garcia-Barrasa J., Lopez-de-Luzuriaga J.M., Monge M. (2011). Silver nanoparticles: Synthesis through chemical methods in solution and biomedical applications. Cent. Eur. J. Chem..

[108] Thakkar K.N., Mhatre S.S., Parikh R.Y. (2010). Biological synthesis of metallic nanoparticles. Nanomedicine.

[109] Leach A.M., McDowell M., Gall K. (2007). Deformation of top-down and bottom-up silver nanowires. Adv. Funct. Mater..

[110] Tak Y., Hong S.J., Lee J.S., Yong K. (2009). Solution-based synthesis of a cds nanoparticle/zno nanowire heterostructure array. Cryst. Growth Des..

[111] Heurlin M., Magnusson M.H., Lindgren D., Ek M., Wallenberg L.R., Deppert K., Samuelson L. (2012). Continuous gas-phase synthesis of nanowires with tunable properties. Nature.

[112] Li S.Z., Huang X., Liu Q., Cao X.H., Huo F.W., Zhang H., Gan C.L. (2012). Vapor-liquid-solid growth of endotaxial semiconductor nanowires. Nano Lett..

[113] Crowell J.E. (2003). Chemical methods of thin film deposition: Chemical vapor deposition, atomic layer deposition, and related technologies. J. Vac. Sci. Technol. A.

[114] Reichelt K., Jiang X. (1990). The preparation of thin films by physical vapour deposition methods. Thin Solid Films.

[115] Humphreys R.G., Satchell J.S., Chew N.G., Edwards J.A., Goodyear S.W., Blenkinsop S.E., Dosser O.D., Cullis A.G. (1990). Physical vapour deposition techniques for the growth of YBa_2_Cu_3_O_7_ thin films. Supercond. Sci. Technol..

[116] Mane R.S., Lokhande C.D. (2000). Chemical deposition method for metal chalcogenide thin films. Mater. Chem. Phys..

[117] Slepička P., Slepičková Kasálková N., Siegel J., Kolská Z., Bačáková L., Švorčík V. (2015). Nano-structured and functionalized surfaces for cytocompatibility improvement and bactericidal action. Biotechnol. Adv..

